# Age‐specific clinical characteristics and outcome in patients over 60 years old with large hemispheric infarction

**DOI:** 10.1002/brb3.1158

**Published:** 2018-11-22

**Authors:** Jie Li, Ping Zhang, Wendan Tao, Xingyang Yi, Jing Zhang, Chun Wang

**Affiliations:** ^1^ Department of Neurology People’s Hospital of Deyang City Deyang China; ^2^ Stroke Clinical Research Unit, Department of Neurology West China Hospital, Sichuan University Chengdu China; ^3^ Department of Neurology Xuanwu Hospital, Capital Medical University Beijing China

**Keywords:** decompressive hemicraniectomy, elderly, large hemispheric infarction, outcome

## Abstract

**Objective:**

We aimed to investigate age‐specific clinical characteristics in patients aged >60 years with large hemispheric infarction (LHI).

**Methods:**

We prospectively enrolled consecutive patients with LHI. Patients were divided into two groups: ≤60 vs. >60 years, and demographics, vascular risk factors, clinical feature, in‐hospital treatment, 3‐month mortality, and unfavorable outcome (defined as a mRS score of 4–6) rate were compared.

**Results:**

Of the 256 cases included, 140 (54.7%) were older than 60 years. Compared with the younger, the older patients had higher rates of hypertension (66.4% vs. 31.0%), coronary heart disease (19.3% vs. 2.6%), atrial fibrillation (53.6% vs. 31.0%; all *p* < 0.001), more history of stroke (21.4% vs. 5.2%, *p* < 0.001), less history of rheumatic heart disease (16.4% vs. 30.1%, *p* = 0.009), and alcohol consumption (12.1% vs. 21.6%, *p* = 0.043). Cardio‐embolism is the most common stroke etiology regardless of age (55.7% and 38.8%, respectively). Furthermore, the elderly less frequently received decompressive hemicraniectomy (4.3% vs. 15.5%, *p* = 0.005) and mechanical ventilation (7.9% vs. 16.4%, *p* = 0.035) and had a higher frequency of stroke‐related complication (83.6% vs. 66.4%, *p* = 0.001). A total of 26 (18.6%) older patients and 15 (12.9%) younger patients died during hospitalization (*p* = 0.221), and 59 (42.1%) older patients and 35 (30.2%) younger patients died at 3 months (*p* = 0.061). Patient aged >60 years had significantly higher unfavorable outcome rate at 3 months (adjusted odds ratio, OR 4.30, 95% confidence interval [CI] 2.08–8.88; *p* < 0.05]. However, older age is not independently associated with 3‐month mortality (42.1% vs. 30.2%, *p* = 0.095 [log‐rank test]).

**Conclusions:**

Large hemispheric infarction patients over 60 years old were a little more than those aged ≤60 years and constitute more than half of those suffered from malignant brain edema and two thirds of in‐hospital death and 3‐month mortality. The elderly had more cardio‐origin risk factors, received less aggressive hospital treatment, and showed higher risk of unfavorable outcome than the younger.

## 1 INTRODUCTION

As we know, aging is the most important independent risk factor for stroke and the incidence of stroke increases significantly with age (Rothwell et al., 2005). It is estimated that the incidence of stroke doubles with each decade of life after the age of 55 years (Sacco et al., 2006). With increasing life expectancy, the world is facing a rapid expansion in its elderly population, especially in developed countries (Wang et al., 2011). Meanwhile, the burden of stroke‐related death and disability is expected to increase together with life expectancy (Kaste, Palomäki, & Sarna, 1995).

Large hemispheric infarction (LHI), which usually refers to total or partial anterior circulation infarct caused by occlusion of internal carotid artery or the proximal middle cerebral artery (MCA), constitutes up to 10% of all supratentorial ischemic strokes (Huttner & Schwab, 2009). LHI is a devastating condition with a high mortality rate of approximately 80% in two intensive care‐based series (Berrouschot, Sterker, Bettin, Köster, & Schneider, 1998; Hacke et al., 1996). Meanwhile, almost no medical treatment has been proven effective (Heiss, 2016). Because of the limitations of medical therapies, decompressive hemicraniectomy (DHC) has been proposed as a therapeutic option for malignant MCA infarction (mMCAi) that is characterized by severe edema and mass effect. Data from pooled analysis of three European randomized trials had demonstrated that early DHC reduced mortality without increasing the risk of severe disability among patients 60 years of age or younger with complete or subtotal space‐occupying MCA infarction (Vahedi et al., 2007). As about 40% of patients with mMCAI are older than 60 years, whether these patients might also benefit from surgery remains unclear (Huttner & Schwab, 2009).

The impact of age on outcome has not yet been well studied in LHI. There are reports of reduced mortality but with poor functional outcome in older patients who undergo DHC in a previous pooled analysis of observational studies and two randomized clinical trials (Gupta, Connolly, Mayer, & Elkind, 2004; Jüttler et al., 2014; Zhao et al., 2012). Older patients have more compensation capacity for space‐occupying stroke; however, those patients tend to have comorbid conditions that are likely to increase the risk of mortality and poor functional outcome. In contrast, younger patients would expect to have better outcome, but a lack of cerebral atrophy may not allow them to tolerate severe edema compared with older patients (Gupta et al., 2004). As risk factors, stroke features were different between younger and older patients (Pohjasvaara, Erkinjuntti, & Vataja, 1997), knowledge of age‐specific characteristics of LHI is essential to establish diagnostic and therapeutic pathways and to set up prevention programs. However, no study is yet available focusing on age‐specific characteristics of patients with LHI over 60 years old.

The objective of the present study is to explore differences in demographics, vascular risk factors, clinical presentation, in‐hospital treatment, and 3‐month outcome in patients with LHI aged >60 years compared with the younger age group in Chinese population.

## 2 METHODS

### 2.1 Study design and subjects

Between 1 October 2011 and 30 September 2014, patients with either a first‐ever stroke or recurrent stroke were registered consecutively after they were admitted to Department of Neurology, People’s Hospital of Deyang City. Data were recorded at the time of assessment using a standardized structured form. Detailed methods for data collection have been previously described (Yi, Lin, & Wang, 2014). In present study, we enrolled patients who were admitted within 30 days from symptoms onset and diagnosed with LHI, which was defined as an ischemic stroke involving more than 50% of the territory of the MCA in computed tomography (CT) scan and/or standard magnetic resonance imaging (MRI), with or without involvement of the adjacent territories (Uhl et al., 2004). All patients had a brain CT scan before initial treatment. A second CT scan or MRI was performed within first 7 days of hospitalization. Other CT scans were performed if patients were suffered neurological deterioration, to identify brain edema or hemorrhagic transformation. We excluded cases with incomplete hospital records or missing imaging that would prevent complete data collection. We also excluded cases with preexisting score of more than 2 on the modified Rankin scale (mRS, a scale of 0–6, with 0 indicating no symptoms and 6 indicating death) and lived dependently (Haan, Limburg, Bossuyt, Meulen, & Aaronson, 1995).

The study protocol was submitted to and approved by the Ethics Committee of People's Hospital of Deyang City. Written informed consent was obtained from all patients before they were enrolled, or from their legal proxies if the patient lost capacity to give the consent.

### 2.2 Data collection and outcome

Baseline data on age, sex, living environment (rural or urban), admission delay, initial stroke severity assessed by the National Institutes of Health Stroke Scale (NIHSS), baseline systolic and diastolic blood pressure, serum glucose on admission, and vascular risk factors were collected. Vascular risk factors investigated in this study included hypertension, diabetes mellitus, dyslipidemia, coronary heart disease, atrial fibrillation, rheumatic heart disease, previous stroke/transient ischemic attack (TIA), current smoking, and alcohol consumption, which have been described in a previous study (Yi et al., 2014). The potential etiology of LHI was classified as large‐artery atherosclerosis, cardio‐embolism, stroke of other determined etiology, and stroke of undetermined etiology according to the Trial of Org 10172 in Acute Stroke Treatment (TOAST) criteria (Adams et al., 1993). In‐hospital treatment analyzed in our study included thrombolysis, DHC, mechanical ventilation, osmotic agents (such as mannitol), antiplatelet agents, anticoagulants, antihypertensives, statins, and antidiabetics. DHC was conducted according to the eligibility and exclusion criteria from the pooled analysis of three European randomized trials (Vahedi et al., 2007), without consideration of the age group, when written informed consent was obtained from the patient or a legal representative. Stroke‐related complications, including both neurological and medical complications during hospitalization, were reviewed by our staff members from hospital records after patient discharge. Neurological complications included brain edema, hemorrhagic transformation, seizures/epilepsy, central hyperthermia, and recurrent stroke, while medical complications during hospitalization included pneumonia, urinary tract infection, gastrointestinal bleeding, electrolyte disturbance, urinary incontinence, acute renal failure, deep venous thrombosis, bedsore, and falls (Johnston et al., 1998).

Patients were followed up at 3‐month after stroke onset by using questionnaires via telephone interview or letter inquiries. The primary outcomes measures in our study were 3‐month mortality and unfavorable outcome (defined as a mRS score of 4–6; Haan et al., 1995).

### 2.3 Statistical analyses

Baseline characteristics were compared between two age groups using student’s *t* tests, Mann–Whitney *U* tests, chi‐square or Fisher exact tests, as appropriate. Univariate analysis was performed to test variables which may affect outcome. The included variables were as follows: (a) age group, (b) NIHSS score on admission, (c) all the risk factors surveyed in our study, (d) in‐hospital treatments, and (e) stroke‐related complication. The odds ratios (ORs) for variables associated with unfavorable outcomes were determined using multivariable logistic regression analyses by the backward stepwise procedure adjusted for variables with *p* < 0.05 on univariate analyses. Cox proportional hazards model was performed to calculate adjusted hazard ratios (HR) of possible influencing factors on 3‐month mortality. Three‐month survival was estimated by Kaplan–Meier method and a log‐rank test was used for survival comparisons between patient groups. All statistical levels quoted are 2‐tailed and a value of *p* < 0.05 was considered significant for all results. The 95% confidence intervals (CIs) were calculated to describe the precision of the estimates. All statistical analysis was performed with SPSS for Windows, version 16.0 (SPSS Inc).

## 3 RESULTS

During the 3‐year study period, 1,542 patients with acute ischemic stroke were consecutively and prospectively registered. Of those, 256 (16.6%) patients with LHI were enrolled in the present study (mean age: 61.6 ± 15.3 years; 133 [52.0%] female). Overall, 140 (54.7%; 55.0% females) patients were aged >60 years (mean age, 72.9 ± 8.1 years; range, 61–99). Of these patients, 119 were aged 60–80 and 21 were over 80 years. Compared with the younger age group, the older patients were less likely to come from the rural area before stroke (25.7% vs. 42.2%, *p* = 0.005) and showed higher level of systolic blood pressure (SBP) on admission (146.3 ± 25.5 vs. 134.0 ± 25.4 mmHg, *p < *0.001). There was no significant difference on baseline NIHSS score between the two groups.

The vascular risk factors in the two age groups are summarized in Table 1. Compared with the younger age group, the older patients had higher rates of hypertension (66.4% vs. 31.0%), coronary heart disease (19.3% vs. 2.6%), atrial fibrillation (53.6% vs. 31.0%), and history of stroke (21.4% vs. 5.2%; all *p* < 0.001). However, older patients had less history of rheumatic heart disease (16.4% vs. 30.1%, *p* = 0.009) and alcohol consumption (12.1% vs. 21.6%, *p* = 0.043) than the younger one. There was no significant difference in the history of TIA between patients of different age group (*p* = 0.895). According to TOAST criteria, the proportion of stroke etiology of LHI was significantly different between the two age groups (*p* = 0.019). The most common stroke subtype in the older age group was cardio‐embolism (55.7%), followed with large‐artery atherosclerosis (22.9%) and stroke of undetermined etiology (17.9%; Table 1).

**Table 1:** Baseline characteristics of patients with LHI between age groups

	Age groups		*p*
	≤60 years (*N* = 116)	>60 years (*N* = 140)	
Age (years)			
Mean ± *SD*	47.9 ± 9.6	72.9 ± 8.1	
Median (range)	50 (15–60)	72 (61–99)	
Female, *n* (%)	56 (48.3)	77 (55.0)	0.284^c^
Rural population, *n* (%)	49 (42.2)	36 (25.7)	0.005^c^
Time from onset (hours), median (range)	24 (1–720)	24 (1–720)	0.067^b^
Baseline NIHSS score, median (range)	14 (4–32)	14 (5–33)	0.825^b^
SBP on admission (mm Hg)	134.0 ± 25.4	146.3 ± 25.5	<0.001^a^
DBP on admission (mm Hg)	82.7 ± 15.8	85.1 ± 16.0	0.235^a^
Baseline serum glucose (mmol/L)	7.7 ± 3.3	7.9 ± 3.4	0.673^a^
Risk factors, *n* (%)			
Hypertension	36 (31.0)	93 (66.4)	<0.001^c^
Diabetes mellitus	19 (16.4)	34 (24.3)	0.120^c^
Dyslipidemia	18 (15.5)	29 (20.7)	0.285^c^
Coronary heart disease	3 (2.6)	27 (19.3)	<0.001^c^
Atrial fibrillation	36 (31.0)	75 (53.6)	<0.001^c^
Rheumatic heart disease	35 (30.2)	23 (16.4)	0.009^c^
Current smoking	31 (26.7)	27 (19.3)	0.157^c^
Alcohol consumption	25 (21.6)	17 (12.1)	0.043^c^
Previous all strokes	6 (5.2)	30 (21.4)	<0.001^c^
Previous TIA	3 (2.6)	4 (2.9)	0.895^c^
Stroke in dominant hemisphere, *n* (%)	62 (53.5)	65 (46.4)	0.263^c^
TOAST classification, *n* (%)			
Large‐artery atherosclerosis	27 (23.3)	32 (22.9)	
Cardio‐embolism	45 (38.8)	78 (55.7)	
Other determined etiology	9 (7.8)	5 (3.6)	
Undetermined etiology	35 (30.2)	25 (17.9)	

DBP: diastolic blood pressure; GCS: Glasgow Coma Scale; NIHSS: National Institutes of Health Stroke Scale; SBP: systolic blood pressure.

a Student *t* test.

b Mann–Whitney *U* test.

c *χ* ^2^ test.

For in‐hospital management of LHI, the older patients more frequently used statins (41.4% vs. 28.5%, *p* = 0.031) in the acute phase of stroke, and less frequently used DHC (4.3% vs. 15.5%, *p* = 0.005) and mechanical ventilation (7.9% vs. 16.4%, *p* = 0.035). There was no significant difference in the use of thrombolysis, antiplatelets, and osmotic agents between the two age groups (*p > *0.05). As for secondary prevention of stroke, the older patients more frequently used antihypertensive (30.0% vs. 15.5%, *p* = 0.006) compared with the younger (Table 2).

**Table 2:** In‐hospital treatment and length of hospital stay of LHI patients between age groups

	Age groups		*p*
	≤60 years (*N* = 116)	＞60 years (*N* = 140)	
In‐hospital Treatments, *n* (%)			
Thrombolysis^a^	5 (4.3)	2 (1.4)	0.250
Decompressive surgery a	18 (15.5)	6 (4.3)	0.002
Mechanical ventilation^a^	19 (16.4)	11 (7.9)	0.035
Osmotic agents^a^	93 (80.2)	112 (80.0)	0.973
Statins in acute phase a	33 (28.5)	58 (41.4)	0.031
Antiplatelets^a^	90 (77.6)	99 (70.7)	0.213
Anticoagulants	8 (6.9)^b^	10 (7.1)^b^	0.939
Antihypertensives	18 (15.5)^b^	42 (30.0)^b^	0.006
Antidiabetic drugs	12 (10.3)^b^	23 (16.4)^b^	0.158
Length of hospital stay (days)			
Mean ± *SD* median (range)	11.3 ± 9.3 9 (1–54)	12.8 ± 11.8 10 (1–78)	0.288^c^ 0.469^d^

a Acute phase treatment.

b Percentage is calculated for patients with indication of the treatment.

c Student *t* test.

d Mann–Whitney *U* test.

The median length of stay in hospital (LOS) was 9 days in the younger age group and 10 days in the older age group. Meanwhile, the mean LOS was 11.3 ± 9.3 and 12.8 ± 11.8 days in the two age groups, respectively. No difference was found on LOS between the two age groups (*p > *0.05). Overall, cases of older age had a higher frequency of stroke‐related complication than the younger (83.6% vs. 66.4%, *p* < 0.001). The most common neurological complication in patients with LHI was malignant brain edema (31.4% in the older age group and 31.0% in the younger age group, respectively), followed with hemorrhagic transformation and seizures/epilepsy. However, there was no significant difference in the events rate of brain edema, hemorrhagic transformation, and seizures/epilepsy between the two age groups (all *p > *0.05). In those patients suffered from malignant brain edema, 55% (44/80) was over 60 years of age. With respect to medical complication, pulmonary infection was the most common medical complication in both groups (62.1% in the older age group and 43.1% in the younger age group, respectively). Nevertheless, cases of older age had a significant higher events rate of pulmonary infection, acute renal failure, and urinary incontinence than those of younger age (all *p* < 0.05; Table 3).

**Table 3:** Stroke‐related complications during hospitalization of LHI patients between age groups

	Age groups		*p*
	≤60 years (*N* = 116)	＞60 years (*N* = 140)	
Complications, *n* (%)	77 (66.4)	117 (83.6)	0.001
Neurological complications, *n* (%)			
Brain edema	36 (31.0)	44 (31.4)	0.946
Hemorrhagic transformation	32 (27.6)	39 (27.9)	0.962
Seizures/epilepsy	12 (10.3)	6 (4.3)	0.059
Central hyperthermia	7 (6.0)	4 (2.9)	0.233
Recurrent stroke	2 (1.7)	1 (0.7)	0.592^a^
Medical complications, *n* (%)			
Pulmonary infection	50 (43.1)	87 (62.1)	0.002
Urinary tract infection	5 (4.3)	14 (10.0)	0.084
Gastrointestinal bleeding	9 (7.8)	21 (15.0)	0.073
Electrolyte disturbance	36 (31.0)	43 (30.7)	0.956
Acute renal failure	4 (3.5)	14 (10.0)	0.041
Urinary incontinence	9 (7.8)	38 (27.1)	<0.001
Bedsore	3 (2.6)	10 (7.1)	0.098
Deep venous thrombosis	6 (5.2)	3 (2.1)	0.307
Falls	(0)	3 (2.1)	0.254^a^

a Fisher exact test.

**Figure 1:**
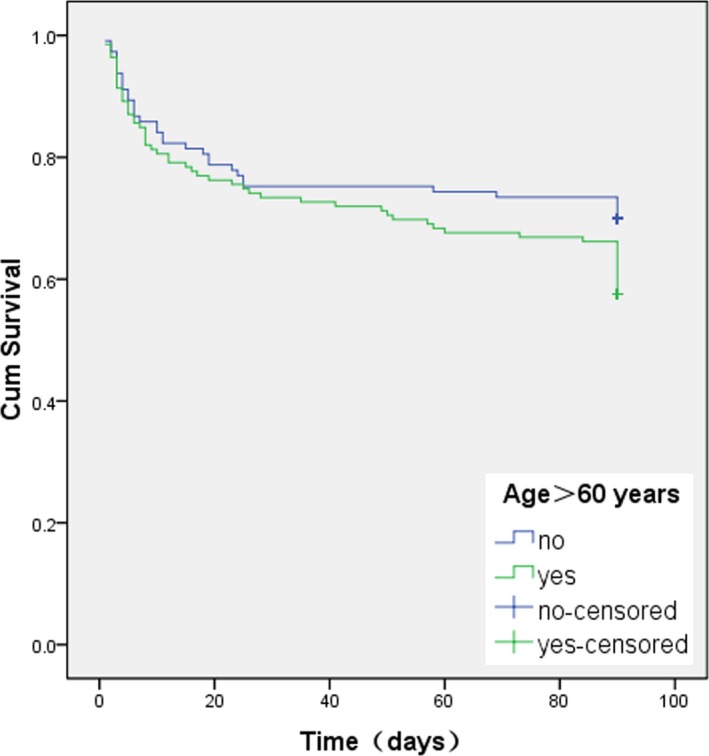
Three‐month survival curves for patients with LHI estimated by Kaplan–Meier Method (patients aged >60 years vs. ≤60 years, *p* = 0.095, log‐rank test)

During hospitalization, 26 (18.6%) patients in the older age group and 15 (12.9%) patients in the younger age group were dead (*p* = 0.221). The most common cause of death during hospitalization was brain herniation in both age groups (19 patients in the older age group and 11 patients in the younger age group, *p* = 0.986). At 3 months, 1.2% (3/256) patients were lost to follow‐up, among which one patient was aged >60 years and two patients were aged ≤60 years, respectively (0.7% vs. 1.7%, *p* = 0.592). A total of 59 (42.1%) patients in the older age group and 35 (30.2%) patients in the younger group died at 3 months (*p* = 0.061). Among those patients, 50% (47/94) were aged 60–80 (Table 4). The most common cause of 3‐month mortality was also brain herniation in both age groups (25 patients in the older age group and 20 patients in the younger age group, *p* = 0.166), and there were more patients over 60 years old died of pulmonary infection at 3 months (18 patients in the older age group and four patients in the younger age group, *p* = 0.035). The 3‐month survival rates of the older patients were not significantly lower than the younger age (*p* = 0.095, log‐rank test; Figure 1). Cox proportional hazards model was employed to determine the independent factors associated with of 3‐month mortality with HR and 95% CI (Table 5). After adjusting for potential confounding factors on 3‐month mortality，age >60 years was eliminated from the model, while baseline NIHSS score (HR 1.11, 95% CI 1.07–1.15), DHC (HR 0.41, 95% CI 0.20–0.87), mechanical ventilation (HR 2.16, 95% CI 1.14–4.12), statins use in acute phase (HR 0.43, 95% CI 0.24–0.75), and stroke‐related complications (HR 11.30, 95% CI 2.74–46.65) were independently associated with 3‐month mortality in LHI patients (all *p < *0.05).

**Table 4:** Clinical outcomes of LHI patients between age groups

	Age groups		*p*
	≤60 years (*N* = 116)	＞60 years (*N* = 140)	
Clinical outcomes, *n* (%)			
In‐hospital death	15 (12.9)	26 (18.6)	0.221
3‐month mortality	35 (30.2)	59 (42.1)	0.061
3‐month unfavorable outcome	46 (39.7)	94 (67.1)	<0.001

**Table 5:** Predictors of 3‐month mortality and poor outcome in LHI patients

Variables	3‐Month mortality		3‐Month poor outcome	
	Univariate analysis	Multivariate analysis^a^	Univariate analysis	Multivariate analysis^b^
Age > 60 years	1.42 (0.93–2.16)		3.09 (1.84–5.17)	4.30 (2.08–8.88)
Baseline NIHSS score	1.14 (1.11–1.18)	1.11 (1.07–1.15)	1.17 (1.11–1.22)	1.16 (1.09–1.22)
Hyperlipidemia	0.35 (0.17–0.73)			
Hypertension			2.28 (1.38–3.79)	2.67 (1.30–5.52)
Atrial fibrillation			1.64 (0.99–2.72)	
Coronary heart disease			2.04 (0.90–4.65)	
Thrombolysis	3.72 (1.51–9.17)			
Decompressive surgery	1.97 (1.12–3.48)	0.41 (0.20–0.87)	10.35 (2.38–45.03)	9.01 (1.50–53.93)
Ventilatory support	3.51 (2.17–5.68)	2.16 (1.14–4.12)	13.88 (3.23–59.65)	
Antiplatelets	0.24 (0.16–0.37)		0.19 (0.08–0.45)	
Statins	0.30 (0.18–0.52)	0.43 (0.24–0.75)	0.39 (0.23–0.67)	0.32 (0.16–0.65)
Stroke‐related complication	19.12 (4.71–77.64)	11.30 (2.74–46.65)	8.47 (4.21–17.02)	3.40 (1.51–7.66)

Notes

Variables which had a significant association with mortality or unfavorable outcome in univariate analysis were listed (*p* < 0.05). Figures in parentheses are 95% confidence intervals (CI).

a Adjusted hazard ratios (HR) with *p* < 0.05 in the Cox proportional hazard regression analysis.

b Adjusted odds ratios (OR) with *p* < 0.05 in the multivariate logistic regression analysis.

Patients in the older age group had a higher rate of unfavorable outcome (mRS Score 4–6) at 90 days (67.1% vs. 39.7%, *p < *0.001; Table 4). Multivariate logistic regression model was employed to determine the independent factors associated with of 3‐month unfavorable outcome with OR and 95% CI (Table 5). After adjusting for potential confounding factors, age >60 years was still the independent factor associated with 3‐month unfavorable outcome (OR 4.30, 95% CI 2.08–8.88; *p* < 0.05). Meanwhile, baseline NIHSS score (OR 1.16, 95% CI 1.09–1.22), hypertension (OR 2.67, 95% CI 1.30–5.52), DHC (OR 9.01, 95% CI 1.50–53.93), statins use in acute phase (OR 0.32, 95% CI 0.16–0.65), and stroke‐related complication (OR 3.40, 95% CI 1.51–7.66) were independently associated with 3‐month unfavorable outcome in LHI patients (all *p *< 0.05).

## 4 DISCUSSION

Up to date, little information is yet available focusing on age‐specific characteristics of LHI patients aged >60 years compared with the younger. In the present study, we found that LHI patients over 60 years old were a little more than those aged ≤60 years (54.7% and 45.3%, respectively), which is a little higher than that reported in previous studies (about 40%–50%; Huttner & Schwab, 2009; Jüttler et al., 2011). It is similar to the data from a randomized trail conducted in Chinese population that included 49 mMCAI patients for DHC and 29 of which (61.7%) were older than 60 years (Zhao et al., 2012). Differences in the proportion of the older between our study and others might be explained by that the elderly were more likely to have cardio‐embolism and total anterior circulation infarct in Asian hospital‐based stroke patients (Chen, Lin, & Po, 2013). Meanwhile, this might also be caused by selection bias since our study was a single‐hospital study. In the present study, 41 (16.0%) patients with LHI died during hospitalization and 94 (36.7%) died at 3 month. We may attribute the lower in‐hospital mortality rate of our cohort in comparison with the 25% mortality rate in a recent case series to the lower age of patients (mean age 61.6 vs. 74.7 years; Arboix et al., 2015). Since almost two thirds of LHI patients died in hospital and at 3 months were over 60 years old, with increasing life expectancy, the proportion of LHI patients aged >60 years tend to rise in the future, which would lead to a heavy burden in China. Therefore, improving outcome of LHI of older age is an important public health problem.

In the present study, we found that LHI of older age was less likely to come from the rural area in our study, this would likely be due to lower rate of rheumatic heart disease (16.4% vs. 30.1%) in the older age group, for most of cases of rheumatic heart disease occur in younger populations living in poverty (White et al., 2010). In our study, the older patients had higher rates of hypertension, coronary heart disease, atrial fibrillation, and more history of stroke. These results fall in line with results of previous study conducted in very old Asians (Chen et al., 2013; Lee, Huang, & Weng, 2007; Wang et al., 2011). Cardio‐embolism is the most common stroke etiology in LHI patients regardless of age, followed with large‐artery atherosclerosis in the older age group. This could be explained by the pathogenesis of occlusion of internal carotid artery or proximal MCA is almost always embolic, either from a proximal cardiac source or atherosclerotic carotid disease (Subramaniam & Hill, 2005). In our study, hypertension and atrial fibrillation were the top two common risk factors of LHI patients especially in the older age group that highlighted the importance of antihypertensive and anticoagulant therapy for the elderly, when regarding the prevention of LHI (Meschia et al., 2014).

In the present study, we found that patients of older age less frequently received DHC and mechanical ventilation (4.3%, 7.9% vs. 15.5%, 16.4%, respectively). This may be due to less active management in older patients (Kaste, Palomäki, & Sarna, 1995; Olindo et al., 2003) and insufficient evidence of DHC in mMCAI patients over 60 years old (Gupta et al., 2004; Jüttler et al., 2014; Zhao et al., 2012). DHC is now recommended for the management of mMCAI patients younger than 60 years of age, but its benefit in older patients is still controversial (Arac, Blanchard, & Lee, 2009; Huttner & Schwab, 2009; Uhl et al., 2004). Meta‐analysis of observational studies indicated that DHC reduced mortality with increasing the risk of poor functional outcome among patients older than 60 years and age may be a crucial factor in predicting functional outcome after DHC in patients with mMCAi (Gupta et al., 2004). DESTINY II trial indicated that DHC increased survival among patients older than 60 years of age with mMCAi, but most survivors were left with disabilities and needed assistance for daily living (Jüttler et al., 2014), which was also confirmed in a small sample randomized trial with Chinese patients (Zhao et al., 2012). Results from the multivariable model of our cohort showed that DHC was a protective factor for 3‐month mortality of LHI patients (HR 0.41; 95% CI, 0.20–0.87). However, the risk of unfavorable outcome was 9‐fold higher than that without receiving DHC after adjusting for potential confounding factors. Nevertheless, a systematic review concluded that despite high rates of physical disability and depression, the majority of patients and/or caregivers are satisfied with life and do not regret having undergone DHC (Rahme, Zuccarello, & Kleindorfer, 2012). As we know, survival with substantial disability instead of death is an outcome that may be acceptable to some patients and caregivers but may not be acceptable to others, especially in older patients. Since 50% of patients died at 90 days were aged 60–80, the decision to perform DHC should be made on an individual basis in every patient older than 60 years of age, especially for patient aged 60–80.

Of note, in our study, mechanical ventilation is not an effective management for reducing 3‐month mortality or unfavorable outcome. More than half of LHI patients receiving mechanical ventilation over 60 years old (6/11) died in hospital, three of the survivors died at 3‐month, and the rest two patients were severely disabled (both mRS score 5). Our finding is consistent with the result of a community‐based study conducted by Mayer et al. (2000), which found that two thirds of stroke patients treated with mechanical ventilation died during hospitalization, and most survivors were profoundly disabled. Meanwhile, there is already some evidence that older age is a considerable predictor of increased mortality in mechanically ventilated stroke patients (Bushnell et al., 1999; Gujjar, Deibert, & Manno, 1998; Steiner et al., 1997). When clinical decision is made for patients with life‐threatening stroke, patients and surrogates should be informed that approximately more than one half of those who are intubated die in hospital and that most of the survivors may remain severely disabled, especially for the elderly.

In the present study, we ascertained cases of older age had a higher frequency of stroke‐related complications and pulmonary infection was the most common complication regardless of age, which resembled the results of previous study in Chinese hospitalized stroke population (Wang et al., 2011). Moreover, LHI patients with stroke‐related complications showed 11.3 times risk of death and 3.4 times risk of unfavorable outcome at 3 months. Since most pulmonary infection and some other stroke‐related complications are potentially preventable or treatable, doctors should pay rigorous attention to the prevention, early detection, and treatment of stroke‐related pneumonia and other complications because of the higher events risk and concomitant poor outcome.

In our study, the most common neurological complication in LHI patients was malignant brain edema, followed with hemorrhagic transformation and seizures/epilepsy. Unexpectedly, there was no significant difference in the events rate of brain edema, hemorrhagic transformation, and seizures/epilepsy between the two age groups, which could partially explain why older age was not associated with in‐hospital death and 3‐month mortality in our cohort. Although previous systematic review about predictors of brain edema in LHI indicated that the mean age of patients with life‐threatening edema was 3.2 years lower than those patients without (Hofmeijer, Algra, & Kappelle, 2008), more than half of patients suffered from brain edema were over 60 years of age in our cohort. This might be partly explained by the higher rate of atrial fibrillation (53.57% vs. 31.03%) and higher proportion of cardio‐embolism (55.71% vs.38.79%) in the older age group, and those patients are more likely to exhibit internal carotid artery or the proximal MCA occlusion which was associated with early recanalization failure，developing severe ischemic stroke and brain edema (Koga et al., 2013; Sakamoto et al., 2014). Although DHC within 48 hr after symptom onset has been proven to benefit highly selected patients with LHI (Huttner & Schwab, 2009), only 0.3% of all ischemic stroke patients would be eligible for DHC on the basis of the strict eligibility criteria in the European hemicraniectomy trials and age >60 years was the most common reason for ineligibility of hemicraniectomy (Rahme, Curry, et al., 2012). Since more than half of patients with mMCAi are older than 60 years, clinical characteristics of those elderly associated with a greater or lesser benefit from DHC require further research.

The results of the present study should be interpreted with caution given its limitations. First, it was a single‐hospital‐based study, with limited generalizability. Some patients with LHI might not be hospitalized, especially those who died before admitted to hospital, so we could not exclude inclusion bias in this study. Second, we only conduct a 3‐month follow‐up so that the long‐term effect of chronic disability remains unclear. Third, since our hospital is one of national comprehensive stroke centers in China, many LHI patients were coming to our hospital by referral from township hospitals and primary stroke centers, and were admitted beyond 48 hr time window, we did not specifically report patients whose LHI was detected within 48 hr after symptom onset. Finally, follow‐up in our study was performed by telephone interview or postal questionnaire instead of a clinic visit which may result in a reporting bias.

## 5 CONCLUSIONS

We identified that LHI patients over 60 years old were a little more than those aged ≤60 years and constitute more than half of those suffered from malignant brain edema and two thirds of in‐hospital death and 3‐month mortality. The elderly had more cardio‐origin risk factors, received less aggressive hospital treatment, and showed higher risk of unfavorable outcome than the younger. Early management of cardiovascular risk should be strengthened in the elderly for prevention of LHI. Meanwhile, since there is significant difference in clinical characteristics, stroke‐related complications, hospital management and outcome between LHI patients over 60 years and the younger, well‐designed randomized trials enrolling the elderly on hospital management, especially on the prevention and treatment of stroke‐related complications, are urgently needed. Furthermore, aggressive hospital treatment such as DHC and mechanical ventilation in the elderly needs more evidence.

## CONFLICT OF INTERESTS

All authors declare that they have no conflict of interests.
